# Practical Model to Optimize the Strategy of Adjuvant Postmastectomy Radiotherapy in T1-2N1 Breast Cancer With Modern Systemic Therapy

**DOI:** 10.3389/fonc.2022.789198

**Published:** 2022-02-24

**Authors:** Fei-Fei Xu, Lu Cao, Cheng Xu, Gang Cai, Shu-Bei Wang, Wei-Xiang Qi, Jia-Yi Chen

**Affiliations:** Department of Radiation Oncology, Ruijin Hospital, Shanghai Jiaotong University School of Medicine, Shanghai, China

**Keywords:** breast cancer, T1-2N1, postmastectomy radiotherapy, risk factors, survival prognosis

## Abstract

**Purpose:**

The effect of adjuvant irradiation after mastectomy in early-stage breast cancer patients remains controversial. The present study aims to explore the clinical benefit obtained from adjuvant radiotherapy among post-mastectomy pT1-2N1 breast cancer patients who received adjuvant modern systemic therapy.

**Methods:**

Medical records of consecutive patients with pT1-2N1 breast cancer who received mastectomy in our institution between January 2009 and December 2016 were retrospectively reviewed. High-risk features consist of patient age, number of positive lymph nodes, T stage, and Ki67 index, which were developed previously at our institution using early-stage breast cancer patients after mastectomy without adjuvant radiotherapy. Differences of survival and local recurrence were compared between no-postmastectomy radiotherapy (PMRT) and PMRT group according to number of risk factors. The time-to-event curves were calculated by the Kaplan–Meier methods and compared by the log-rank test. Propensity score matching (PSM) was performed to reduce the imbalances in patient characteristics.

**Results:**

A total of 548 patients were enrolled (no-PMRT: 259 and PMRT: 289). After a median follow-up of 69 months, the 5-year rate of DFS, BCSS, and LRR in the overall cohort was 90.2%, 97.4%, and 3.6%, respectively. PMRT did not significantly improve DFS, BCSS, and LRRFS in the whole cohort. Patients were divided into low-risk (with no or one risk factor) and high-risk (with two or more risk factors) groups. According to the univariable and multivariable analysis, high-risk group (HR = 1.81, 95% CI 1.11–2.98, *p* = 0.02) was demonstrated as an independent risk factor for DFS. For the high-risk group, PMRT significantly improved DFS from 81.4% to 91.9% and BCSS from 95.5% to 98.6% and decreased the 5-year rate of LRR from 5.6% to 1.4%, respectively (*p* < 0.01, *p* = 0.05, and *p* = 0.06). However, no survival benefit from PMRT was observed in the low-risk group in terms of DFS, BCSS, and LRR (*p* = 0.45, *p* = 0.51, and *p* = 0.99, respectively). In multivariate analysis, PMRT remained an independent prognostic factor for DFS (HR = 0.50, 95% CI 0.24–1.00, *p* = 0.05) in the high-risk group. After PSM analysis, the survival benefit of PMRT was sustained in high-risk patients.

**Conclusion:**

PMRT significantly improved DFS in high-risk pT1-2N1 breast cancer patients, but not in low-risk patients. Independent validation of our scoring system is recommended.

## Introduction

Globally, breast cancer is the most commonly diagnosed cancer in women with a growing trend in both incidence and mortality (1). On the molecular level, breast cancer is a heterogeneous disease, which could be categorized into subtypes mainly based on the presence or absence of molecular markers for human epidermal growth factor receptor 2 (HER2) and hormone receptors [HR, including estrogen receptor (ER) and progesterone receptor] and/or BRCA mutations (2). As a result, a multidisciplinary team is recommended to determine the optimal locoregional (surgery and radiation therapy) and systemic management strategies for breast cancer. Modern systemic therapies, including endocrine therapy for HR-positive disease, anti-HER2 therapy for HER2-positive disease, chemotherapy based on anthracycline and taxane, bone-stabilizing agents, poly (ADP-ribose) polymerase inhibitors for BRCA mutation carriers, and immunotherapy, have been demonstrated to significantly improve the survival outcomes of breast cancer patients (3). Post-mastectomy radiotherapy (PMRT) is an important local treatment for breast cancer with microscopic residual disease. In general, the indications for PMRT were strongly recommended for breast cancer involving a tumor size of >5 cm, presence of more than three positive lymph nodes, or positive surgical margins (4).

However, the role of PMRT in pT1-2N1 breast cancer patients remains debated in daily clinic (5). An updated report from the Early Breast Cancer Trialists’ Collaborative Group (EBCTCG) in 2014 confirmed that adjuvant PMRT significantly reduced both recurrence and breast cancer mortality in the women with one to three positive lymph nodes (6, 7). However, this meta-analysis has been criticized for its limitations, mainly less intensive systemic therapy, limited axillary dissection in some trials, and the sub-optimal radiation techniques. The high locoregional recurrence (LRR) of 20.3% at 10 years in EBCTCG meta-analysis is also quite far from the LRR rated reported in later trials (8). In addition, the clinical benefit obtained from PMRT significantly varies with primary tumor size and number of positive lymph node. A recent study from University of Chicago showed that PMRT improved the survival prognosis among patients with 3 positive lymph nodes and tumors 2–5 cm in size, but no beneficial effect for patients with 1–2 positive nodes and tumors 2 cm in size or smaller (9). Thus, investigating risk factors is critically important to identify early-stage breast cancer patients who might benefit from PMRT after mastectomy (4, 10).

A number of risk factors for survival in early breast cancer patients have been reported, but the results are controversial (11). Prior to the present study, we have established a nomogram for predicting the prognosis of patients with pN0-1 breast cancer who were treated with mastectomy and without adjuvant radiotherapy (12). The model was externally validated in an independent cohort of 1,356 patients from one phase III trial (NCT00041119). Finally, pathological T stage, number of positive lymph nodes, age, and Ki67 index were found to be significant predictors for breast cancer specific survival (BCSS) in post-mastectomy breast cancer with pN0-1. In the present study, we aim to validate whether the practical prognostic scoring system based on these four risk factors in our previous study can identify high-risk pT1-2N1 breast cancer patients who could benefit from PMRT.

## Materials and Methods

### Patients’ Selection

From January 2009 to December 2016, a total of 642 consecutive newly diagnosed invasive breast cancer patients undergoing mastectomy and sentinel lymph node biopsy or axillary lymph node dissection with pathological T1-2N1 were identified at our institution. Ninety-four patients were excluded from the present analysis for the following reasons: (1) neoadjuvant chemotherapy; (2) lack of information about tumor size, pathological type, Ki67 index, and radiotherapy; (3) pathologically diagnosed as ductal carcinoma i*n situ*, lobular carcinoma *in situ*, or Paget’s disease. Finally, 548 patients were enrolled for analysis in the present study.

### Adjuvant Radiotherapy

For patients treated with adjuvant PMRT, dose prescription to the chest wall (CW) and regional nodes (supraclavicular, infraclavicular with or without internal mammary lymph nodes) was 50 Gy in 25 fractions. CW irradiation was given using field-in-field forward-planned intensity-modulated radiotherapy using photons and regional nodes were treated using an anterior mixed photon and electron beam. The volume delineation and definition were determined according to the Radiation Therapy Oncology Group (RTOG) guidelines (13).

### Outcome’s Definitions

Disease-free survival (DFS) was defined as the time from surgery to the time of the first recurrence in the ipsilateral chest wall or in regional nodal or distant sites or death from any cause. BCSS was defined as the time from surgery till death of breast cancer. LRR was defined as the time from surgery to the time of a first recurrence in the ipsilateral chest wall or in the ipsilateral regional nodal (including axillary, supraclavicular, infraclavicular, and internal mammary lymph nodes). Follow-up time was calculated from the date of surgery to the first event or last confirmed date of breast cancer disease-free status.

### Statistical Analysis

For categorical variables, differences between the no-PMRT and PMRT groups were evaluated by using Pearson’s chi square statistics. The time-to-event curves were calculated by the Kaplan–Meier methods and compared by the log-rank test. Hazard ratios (HRs) and corresponding 95% CIs were estimated using the Cox proportional hazards regression model. Given the difference between patients with and without PMRT, PSM was applied to balance measurable confounders. Patients were matched based on their estimated propensity using 1:1 matching *via* nearest method without replacement with a caliper of 0.05. All statistical tests were two-sided and *p* < 0.05 was considered significant. The software package SPSS 24.0 (IBM corporation, USA) was used for analysis.

## Results

### Baseline Characteristics

In total, 548 patients who received mastectomy and were diagnosed as pT1-2N1 breast cancer were enrolled. A total of 289 patients were treated with adjuvant PMRT, and all completed scheduled radiotherapy. The baseline characteristics of these patients are listed in **Table 1**. The median age at diagnosis was 56 years (range, 28–91). The median tumor size was 2.5 cm (range, 0.3–5.0) in the whole cohort. Among 455 patients who received adjuvant chemotherapy, 86.6% received anthracycline and taxane-based chemotherapy. In ER-positive patients, 89.6% received the endocrine therapy. Anti-HER2 therapy was given to 62.9% of HER2-positive patients.

**Table 1:** Patient and treatment characteristics.

Characteristics	Whole cohort (*N* = 548)	No-PMRT (*N* = 259)	PMRT (*N* = 289)	*p*-value
**Age (years)**				<0.01
Median (range)	56 (28–91)	58 (29–91)	54 (28–78)	
≤40	50 (9.1)	14 (5.4)	36 (12.5)	
>40	498 (90.9)	245 (94.6)	253 (87.5)	
**Menopausal status**				0.09
Premenopausal	198 (36.1)	84 (32.4)	119 (39.4)	
Postmenopausal	350 (63.9)	175 (67.6)	179 (60.6)	
**Tumor size (cm)**				0.09
Median (range)	2.5 (0.3–5.0)	2.0 (0.5–5.0)	2.5 (0.3–5.0)	
≤2.0	254 (46.4)	130 (50.2)	124 (42.9)	
2.0-5.0	294 (53.6)	129 (49.8)	165 (57.1)	
**Nuclear grade**				0.05
Low-Intermediate	282 (57.1)	146 (61.6)	136 (52.9)	
High	212 (42.9)	91 (38.4)	121 (47.1)	
Unknown	58	23	35	
**Axillary surgery**				0.01
SLNB alone	12 (2.2)	10 (3.9)	2 (0.7)	
ALND	536 (97.8)	249 (96.1)	287 (99.3)	
**Number of resected LN**	16 (2–35)	16 (2–35)	15 (4–34)	0.86
**Number of positive LN**				<0.01
1–2	450 (82.1)	226 (87.3)	224 (77.5)	
3	98 (17.9)	33 (12.7)	65 (22.5)	
**ER status**				<0.01
Positive	416 (76.6)	217 (84.1)	199 (69.8)	
Negative	127 (23.4)	41 (15.9)	86 (30.2)	
Unknown	5	1	4	
**Ki67 index**				<0.01
≤20%	203 (37.0)	112 (43.2)	91 (31.5)	
>20%	345 (63.0)	147 (56.8)	198 (68.5)	
**HER2 status**				<0.01
Positive	129 (23.7)	48 (18.6)	81 (28.3)	
Negative	415 (76.3)	210 (81.4)	205 (71.7)	
Unknown	4	1	3	
**Molecular subtype**				<0.01
Luminal	416 (76.6)	217 (83.8)	199 (69.8)	
HER2 positive	62 (11.4)	18 (7.0)	44 (15.4)	
Triple negative	65 (12.0)	23 (8.9)	42 (14.7)	
Unknown	5	1	4	
**Chemotherapy**				<0.01
Yes	455 (86.2)	193 (77.2)	262 (94.2)	
No	73 (13.8)	57 (22.8)	16 (5.8)	
Unknown	20	9	11	
**Target therapy in HER2 positive (*n* = 129)**				0.46
Yes	78 (62.9)	27 (58.7)	51 (65.4)	
No	46 (37.1)	19 (41.3)	27 (34.6)	
Unknown	5	2	3	
**Endocrine therapy in ER positive (*n* = 416)**				0.10
Yes	353 (89.6)	184 (87.2)	169 (92.3)	
No	41 (10.4)	27 (12.8)	14 (7.7)	
Unknown	22	6	16	

As shown in **Table 1**, patients in the PMRT group had more risk factors including younger age, larger tumor, more axillary lymph nodes involved, and unfavorable biomarkers. Accordingly, higher portion of patients received chemotherapy in the PMRT group (*p* < 0.01).

### Survival Outcomes in Overall Cohort and Different Subgroups

After a median follow-up of 69 months (range, 2–128), 7 patients developed LRR only, 37 patients had distant metastasis only, and 13 patients developed LRR and distant metastasis. A total of 32 patients died in the entire cohort, with 23 attributed to breast cancer. The 5-year rate of DFS and BCSS was 90.2% and 97.4%, respectively. The 5-year rate of LRR was 3.6%.

**Figure 1:**
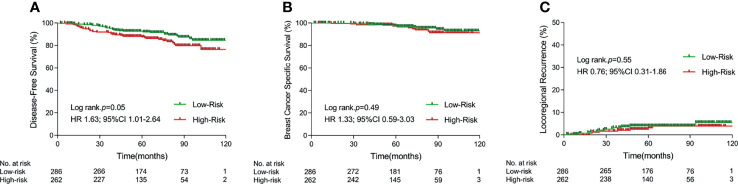
Kaplan–Meier curves for 5-year disease-free survival **(A)**, 5-year breast cancer specific survival **(B)**, and 5-year locoregional recurrence **(C)** in different risk cohorts. (PMRT, postmastectomy radiotherapy).

Four risk parameters, established and validated by our previous study to be independent risk factors for predicting BCSS in pN0-1 breast cancer patients receiving mastectomy, namely, age (≤40 versus >40 years old), number of positive lymph nodes (1–2 versus 3 positive lymph nodes), T stage (T1 versus T2), and Ki67 index (≤20% versus >20%), were utilized to divide patients into a low-risk group, which was defined as patients with no or one risk factor, and a high-risk group, which was defined as patients with two or more risk factors. There were 286 and 262 patients in the low-risk group and high-risk group, respectively, in which 127 and 162 patients received PMRT, respectively. Five-year rates of DFS, BCSS, and LRR were 92.6% versus 87.5% (*p* = 0.05), 97.5% versus 97.2% (*p* = 0.49), and 4.1% versus 3.2% (*p* = 0.55) between the low- and high-risk subgroups, respectively (shown in **Figure 1**).

### Univariate and Multivariate Analysis for Survival Outcomes

In the whole cohort, chemotherapy (Yes vs. No) and risk group (high risk vs. low risk) were found to be significant prognostic factors for DFS (*p* < 0.01 and *p* = 0.04, respectively) by univariate analysis. By multivariate analysis, no chemotherapy (HR = 2.69, 95% CI 1.51–4.79, *p* < 0.01) and high-risk group (HR = 1.81, 95% CI 1.11–2.98, *p* = 0.02) remained independent risk factors for DFS. The detailed univariable and multivariable analysis for DFS is shown in **Table 2**.

**Table 2:** The univariate and multivariable analyses for outcomes.

Characteristics	DFS					
	Univariate analyses			Multivariable analyses		
	*N* of event	5-year rate	*p*-value	HR	95% CI	*p-*value
**Age (years)**			0.59			
≤40	7	89.2				
>40	61	90.3				
**Menopausal status**			0.16			
Premenopausal	19	93.6				
Postmenopausal	49	88.7				
**Tumor size (cm)**			0.06			
≤2.0	25	94.2				
2.0–5.0	43	88.2				
**Nuclear grade**			0.37			
Low-Intermediate	33	91.7				
High	30	87.6				
**Axillary surgery**			0.85			
SLNB alone	1	90.9				
ALND	67	90.5				
**Number of positive LN**			0.38			
1–2	53	90.5				
3	15	89.1				
**ER status**			0.51			
Positive	17	86.8				
Negative	51	91.1				
**Ki67 index**			0.38			
≤20%	20	92.9				
>20%	48	88.9				
**HER2 status**			0.36			
Positive	19	90.6				
Negative	49	88.6				
**Molecular subtype**			0.39			
Luminal	51	91.1				
HER2 positive	10	84.6				
Triple negative	7	88.7				
**Chemotherapy**			<0.01			
Yes	51	91.0		1		
No	16	84.5		2.69	1.51–4.79	<0.01
**Target therapy**			0.18			
Yes	13	86.9				
No	54	90.6				
**Endocrine therapy**			0.28			
Yes	45	91.8				
No	22	85.7				
**PMRT**			0.13			
Yes	26	91.7				
No	42	88.8				
**Subgroups**			0.04			
Low risk	29	92.6		1		
High risk	39	87.5		1.81	1.11–2.98	0.02

### Survival Benefits From PMRT in Different Risk Groups

**Figure 2:**
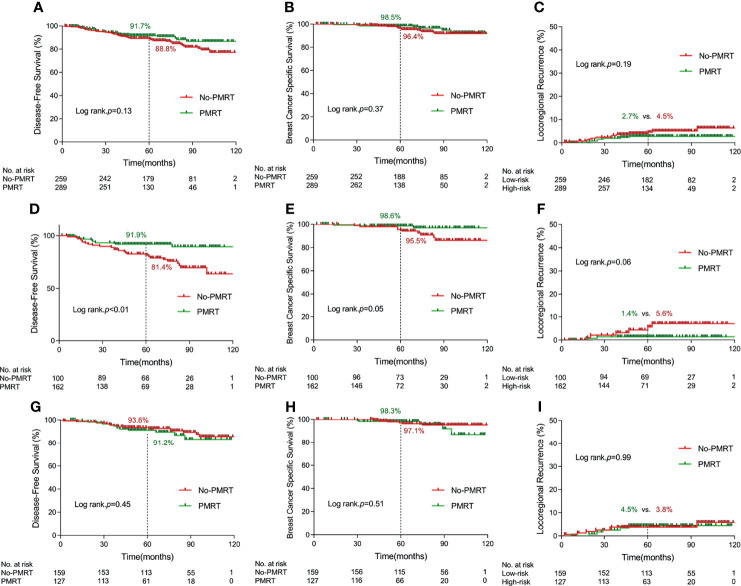
Kaplan–Meier curves for 5-year disease-free survival, 5-year breast cancer specific survival, and 5-year locoregional recurrence according to delivery of postmastectomy radiotherapy in terms of different cohorts. [**(A–C)** in the whole cohort; **(D–F)** in the high-risk subgroup; **(G–I)** in the low-risk group] (PMRT, postmastectomy radiotherapy).

After a median follow-up of 69 months (range 2–128), 8 and 15 breast cancer deaths occurred in the PMRT and no-PMRT group, respectively. No significant difference was found between PMRT and no-PMRT groups in terms of 5-year rate of DFS (91.7% vs. 88.8%, *p* = 0.13, **Figure 2A**), BCSS (98.5% vs. 96.4%, *p* = 0.37, **Figure 2B**), and LRR (2.7% vs. 4.5%, *p* = 0.19, **Figure 2C**).

For the high-risk group, the Kaplan–Meier survival analysis indicated that PMRT significantly improved 5-year rate of DFS from 81.4% to 91.9% (*p* < 0.01, **Figure 2D**), BCSS from 95.5% to 98.6% (*p* = 0.05, **Figure 2E**), and LRR from 5.6% to 1.4% with marginal significance (*p* = 0.06, **Figure 2F**). For the low-risk group, there was no significant difference in DFS, BCSS, and LRR between PMRT and no-PMRT patients (**Figures 2G–I**). By multivariate analysis, chemotherapy and PMRT remained independent prognostic factors for DFS (HR = 0.27, 95% CI 0.12–0.58, *p* < 0.01 and HR = 0.50, 95% CI 0.24–1.00, *p* = 0.05, respectively) in the high-risk group. The results of univariate and multivariate survival analysis in the high-risk and low-risk groups separately are detailed in **Supplementary Table 1**.

Since the baseline characteristic significantly varied between the PMRT group and no-PMRT group, we performed PSM to reduce the potentially selection bias. After PSM analysis, a total of 392 matched patients were finally included for analysis. No significant difference was observed between PMRT and no-PMRT groups in the overall cohort (shown in **Supplementary Table 2**). Similarly, the 5-year rate of DFS in patients treated with PMRT was comparable to those who did not receive PMRT (90.6% vs. 88.5%, *p* = 0.36) in overall matched cohort. Consistent with previous results, patients with more than two high-risk factors remained a poor independent risk factor for DFS in multivariate analysis (HR = 1.81, 95% CI 1.05–3.11, *p* = 0.03). In addition, survival benefit obtained from PMRT remained significant among breast cancer with more than two high-risk factors after PSM (5-year rate of DFS: 92.3% vs. 82.1%, *p* = 0.03), while no significant survival benefit from PMRT was observed among patients presented with less than one low-risk factor after PSM (5-year rate of DFS: 89.1% versus 93.6%, *p* = 0.29).

In conclusion, our results were consistent before or after PSM, which further confirmed that the high-risk group, defined as patients with two or more risk factors, was an independent risk factor for DFS, and PMRT should be recommended for patients in that population.

## Discussion

In this study, we investigated the impact of PMRT on survival outcomes among patients with T1-2N1 breast cancer treated with modern systematic therapies by using a scoring system composed of 4 clinical–pathological risk factors. Based on the number of risk factors, we divided patients into two risk groups [low risk (0–1 risk factor) vs. high-risk (≥2 risk factors)]. In the multivariate analysis, we found that the high-risk group and chemotherapy were two independent risk factors for DFS and survival benefit of PMRT was limited to the high-risk group only.

The trend in the receipt of PMRT in patients with T1-2N1 breast cancer has varied significantly over years and utilization of PMRT has increased from 14.1% to 23.5% in Asia in recent years (14). Nevertheless, significant controversy remains regarding the benefit of PMRT or regional nodal irradiation (RNI) in this population. Prior to the present study, the DBCG 82 b&c randomized trials demonstrated lower risk of LRR and better survival outcomes with the addition of PMRT (15). However, the higher risk of LRR and suboptimal BCSS have been attributed to the less than standard systemic therapy and axillary surgery in the DBCG trials. Subsequently, McBride et al. investigated the clinical benefit of PMRT among patients with T1-2N1 breast cancer and treated with mastectomy and modern systemic treatment, but no significant difference in 5-year LRR was observed (16). Another study performed by Muhsen et al., which recruited 1,087 patients with pT1-2N1 breast cancer, found no survival benefit from PMRT (17). A large sample analysis from the surveillance, epidemiology, and end results program (SEER) data also found that the survival outcomes were comparable between PMRT and no-PMRT patients in the modern era (14). Consistent with previous results, we also found that in the general population of T1-2N1 breast cancer patients, the clinical benefit from PMRT in the era of modern systemic therapy was not significant. In our cohort, adjuvant chemotherapy was prescribed to 86.2% of included patients and up to 88.5% of patients had ≥10 axillary lymph nodes removed. As a result, in the era of modern systemic therapy with adjuvant chemotherapy typically containing anthracycline and taxanes, higher proportion of HER2-positive patients receiving anti-HER2 therapy, and standard adjuvant hormonal therapy in HR-positive patients, the risk of tumor recurrence has been significantly decreased. Therefore, establishing a risk scoring system is critically important to identify early-stage breast cancer patients who might benefit from PMRT after mastectomy.

A practical reference risk stratifying system appears to be essential to identify patients who would benefit most from PMRT at the present time. A number of risk factors that have been identified by nomograms combining different risk factors have been developed as well (18, 19). The most representative risk factors identified were patient age, number of positive lymph nodes, histological grade, and lympho-vascular invasion (20). However, the absolute risk of LRR and survival benefit from PMRT with regard to risk stratifying system in T1–2N1 patients after mastectomy remain heterogeneous. Data from SEER population claimed that the benefit of PMRT was observed in patients with high-risk (2 or 3 positive nodes with tumors 2–5 cm in size) but not in patients with low-risk disease (1 or 2 positive nodes with tumors <2 cm in size) (9). A more recent retrospective study by Park et al. found that close resection margin was the only independent factor for worse prognosis among post-mastectomy patients undergoing modern systematic therapies (21). Molecular subtypes play a critical part in the decision-making of systemic therapy, but its role in tailoring local–regional radiotherapy remains undefined, even though ongoing studies aim to explore this. The TAILOR RT trial sponsored by the Canadian Cancer Trials Group is investigating the role of PMRT in favorable patients with one to three positive axillary nodes who have ER-positive tumors with low-risk Oncotype DX recurrence scores (NCT03488693). Our analysis failed to recognize the molecular subtypes as a significant prognostic factor of DFS by univariate analysis and multivariate analysis. One possible reason might be the improvement of patients’ survival with the application of comprehensive systemic therapy with almost 86.2% of patients receiving 4–8 cycles of chemotherapy of anthracycline or taxane, 62.9% of patients receiving anti-HER2 therapy in HER2-positive subtypes, and 89.6% patients receiving endocrine therapy in ER-positive subtypes in our study. Consistent with our results, a large sample study, which enrolled 1,474 postmastectomy patients staged pT1-2N1 between 2006 and 2012, showed that molecular subtypes also failed to significantly influence the survival and local prognosis with application of optimal systemic therapy (98.1% with anthracycline or taxane chemotherapy, 95.1% with hormonal therapy in HR-positive patients, and 47.4% with anti-HER2 therapy in HER2-positive patients) (22). As yet, the molecular subtype is not included in National Comprehensive Cancer Center (NCCN) guidelines to guide the decision-making of PMRT in this population (23). Results of prospective trials are still of great importance to define the role of molecular subtypes and other more elaborate biomarkers in the decision-making of radiotherapy.

In our previous study, patients with positive or close surgical margin were excluded while age, number of positive lymph nodes, tumor size, and Ki67 index remained as independent risk factors for BCSS in T1-2N0-1 breast cancer (12). Our results showed that benefits from PMRT were disparate between different risk groups. PMRT significantly improved DFS in the high-risk group with 2–4 risk factors while it did not improve in the low-risk group with 0–1 risk factor. Although it was a single-center experience, it provided a basis to conduct a multiple institutional study in a second phase. The randomized SUPREMO trial was prospectively designed to evaluate the role of PMRT in 1,688 women with intermediate-risk breast cancer defined as T1-2N1, T3N0, or T2N0 with lympho-vascular invasion and high grade who underwent mastectomy between 2006 and 2013 (24, 25). The results of this study are expected by the end of 2023.

Recent studies incorporating information on the molecular profile of breast cancer aim to further tailor radiotherapeutic decisions based on risk stratification and potentially intrinsic radiosensitivity of different subtypes. Shao et al. conducted a retrospective cohort-based study and demonstrated that among patients with high-risk factors (T2 stage and 3 positive lymph nodes disease), PMRT prolonged over-survival only in the Luminal A subtype, but not for the triple-negative and HER2-positive subgroups (26). In addition to those known prognostic biomarkers, genomic profile will provide additional prognostic information to risk stratification. However, most of these studies were retrospective; thus, the evidence was relatively low. An observational cohort study using data from the American National Cancer Database (NCDB) and SEER found that the improved survival associated with PMRT was limited to patients with a low Oncotype DX recurrence score (RS) (27). Others had reported that RS could not define the patients who will benefit from PMRT or not (28). Mamounas et al. though found that a high RS predicted a higher risk of LRR in general, while such association was not established when N1 patients receiving mastectomy were further analyzed (29). At the present time, majority of the panel of 2021 SG-BCC agree that commercially available multigene signatures (e.g., MammaPrint and Recurrence Score) should not provide a solid recommendation for deciding RNI (92%) or PMRT (89%) when prospective trials such as TAILOR RT are still ongoing (30). Most of the ongoing trials integrating genomic profile are focused on ER-positive, HER2-negative tumors with one to three positive axillary nodes (31). To acknowledge the advantage of molecular and genomic profile in individualizing risk in a defined population, the inconvenience that other molecular subtypes are not covered by most of the trials should also be noticed. While awaiting these results, our present analysis provides a practical model of available clinical–pathological information and biomarkers in consideration of an individualized PMRT.

There are limitations of this study that need to be mentioned. First, this is a retrospective study of our institute; thus, potential selection bias could not be excluded. Second, the median follow-up of 69 months is relatively limited, which might underestimate the actual survival outcomes of this patient population.

In summary, our retrospective study provided a practical model to optimize the triage of PMRT in a highly debatable population, T1-2N1 breast cancer patients. The risk scoring system composed of four clinical–pathological risk factors can be applied to identify the high-risk patients who might benefit from PMRT undergoing modern systemic adjuvant therapy.

## Data Availability Statement

The original contributions presented in the study are included in the article/**Supplementary Material**. Further inquiries can be directed to the corresponding authors.

## Ethics Statement

The studies involving human participants were reviewed and approved by the Ethical Committee of Ruijin Hospital Affiliated Medicine School of Shanghai Jiao Tong University. Written informed consent for participation was not required for this study in accordance with the national legislation and the institutional requirements.

## Author Contributions

Concept, design, analysis and interpretation of data, and manuscript writing: F-FX, W-XQ, and J-YC. Collection of data and final approval of manuscript: F-FX, LC, CX, GC, S-BW, W-XQ, and J-YC. All authors contributed to the article and approved the submitted version.

## Funding

This study was supported in part by the National Key Research and Development Program of China (grant numbers 2016YFC0105409), Clinical Research Plan of SHDC (grant numbers SHDC2020CR2052B and SHDC2020CR4070), Shanghai Municipal Education Commission-Gaofeng Clinical Medicine Grant Support (grant numbers 20171904), National Natural Science Foundation of China (grant numbers 81673102, 81602791, 81702601, 81803164, and 81972963), Shanghai Jiaotong University Translational Medicine Fund Support (grant numbers ZH2018QNA54), Special Construction of Integrated Chinese and Western Medicine in General Hospital (grant numbers ZHYY-ZXYJHZ X-2-201704 and ZHYY-ZXYJHZ X-2-201913), and Scientific and Technological Innovation Action Plan of Shanghai Science and Technology Committee (grant numbers 19411950900 and 19411950901).

## Conflict of Interest

The authors declare that the research was conducted in the absence of any commercial or financial relationships that could be construed as a potential conflict of interest.

The handling editor YC is currently organizing a Research Topic with the author JC.

## Publisher’s Note

All claims expressed in this article are solely those of the authors and do not necessarily represent those of their affiliated organizations, or those of the publisher, the editors and the reviewers. Any product that may be evaluated in this article, or claim that may be made by its manufacturer, is not guaranteed or endorsed by the publisher.

## References

[1] Bray F Ferlay J Soerjomataram I Siegel RL Torre LA Jemal A. Global Cancer Statistics 2018: GLOBOCAN Estimates of Incidence and Mortality Worldwide for 36 Cancers in 185 Countries. CA: Cancer J Clin (2018) 68(6):394–424. doi: 10.3322/caac.21492 30207593

[2] Waks AG Winer EP. Breast Cancer Treatment: A Review. JAMA (2019) 321(3):288–300. doi: 10.1001/jama.2018.19323 30667505

[3] Harbeck N Penault-Llorca F Cortes J Gnant M Houssami N Poortmans P. Breast Cancer. Nat Rev Dis Primers (2019) 5(1):66. doi: 10.1038/s41572-019-0111-2 31548545

[4] Recht A Comen EA Fine RE Fleming GF Hardenbergh PH Ho AY. Postmastectomy Radiotherapy: An American Society of Clinical Oncology, American Society for Radiation Oncology, and Society of Surgical Oncology Focused Guideline Update. Pract Radiat Oncol (2016) 6(6):e219–e34. doi: 10.1016/j.prro.2016.08.009 27659727

[5] Chagpar AB. Debate: Postmastectomy Radiation Therapy in T1/2N1 Disease. Ann Surg Oncol (2021) 28(10):5456–60. doi: 10.1245/s10434-021-10500-5 34324110

[6] McGale P Taylor C Correa C Cutter D Duane F Ewertz M. Effect of Radiotherapy After Mastectomy and Axillary Surgery on 10-Year Recurrence and 20-Year Breast Cancer Mortality: Meta-Analysis of Individual Patient Data for 8135 Women in 22 Randomised Trials. Lancet (London England) (2014) 383(9935):2127–35. doi: 10.1016/s0140-6736(14)60488-8 PMC5015598 24656685

[7] Kayali M Abi Jaoude J Tfayli A El Saghir N Poortmans P Zeidan YH. Post-Mastectomy Radiation Therapy in Breast Cancer Patients With 1-3 Positive Lymph Nodes: No One Size Fits All. Crit Rev Oncol Hematol (2020) 147:102880. doi: 10.1016/j.critrevonc.2020.102880 32045847

[8] Moo TA McMillan R Lee M Stempel M Patil S Ho A. Selection Criteria for Postmastectomy Radiotherapy in T1-T2 Tumors With 1 to 3 Positive Lymph Nodes. Ann Surg Oncol (2013) 20(10):3169–74. doi: 10.1245/s10434-013-3117-0 23975289

[9] Huo D Hou N Jaskowiak N Winchester DJ Winchester DP Yao K. Use of Postmastectomy Radiotherapy and Survival Rates for Breast Cancer Patients With T1-T2 and One to Three Positive Lymph Nodes. Ann Surg Oncol (2015) 22(13):4295–304. doi: 10.1245/s10434-015-4528-x 25820998

[10] Cardoso F Kyriakides S Ohno S Penault-Llorca F Poortmans P Rubio IT. Early Breast Cancer: ESMO Clinical Practice Guidelines for Diagnosis, Treatment and Follow-Up. Ann Oncol (2019) 30(10):1674. doi: 10.1093/annonc/mdz189 31236598

[11] Bazan JG Majithia L Quick AM Wobb JL Terando AM Agnese DM. Heterogeneity in Outcomes of Pathologic T1-2n1 Breast Cancer After Mastectomy: Looking Beyond Locoregional Failure Rates. Ann Surg Oncol (2018) 25(8):2288–95. doi: 10.1245/s10434-018-6565-8 29916008

[12] Qi WX Cao L Xu C Zhao S Chen J. Established and Validated Novel Nomogram for Predicting Prognosis of Post-Mastectomy Pn0-1 Breast Cancer Without Adjuvant Radiotherapy. Cancer Manage Res (2021) 13:3517–27. doi: 10.2147/cmar.s292233 PMC8079251 33935517

[13] White J Tai A Arthur D Buchholz T MacDonald S Marks L. Breast Cancer Atlas for Radiation Therapy Planning: Consensus Definitions. Book Breast Cancer Atlas for Radiation Therapy Planning. (2009).

[14] Li FY Lian CL Lei J Wang J Hua L He ZY. Real-World Impact of Postmastectomy Radiotherapy in T1-2 Breast Cancer With One to Three Positive Lymph Nodes. Ann Transl Med (2020) 8(7):489. doi: 10.21037/atm.2020.03.49 32395533 PMC7210210

[15] Danish Breast Cancer Cooperative G Nielsen HM Overgaard M Grau C Jensen AR Overgaard J. Study of Failure Pattern Among High-Risk Breast Cancer Patients With or Without Postmastectomy Radiotherapy in Addition to Adjuvant Systemic Therapy: Long-Term Results From the Danish Breast Cancer Cooperative Group DBCG 82 B and C Randomized Studies. J Clin Oncol (2006) 24(15):2268–75. doi: 10.1200/JCO.2005.02.8738 16618947

[16] McBride A Allen P Woodward W Kim M Kuerer HM Drinka EK. Locoregional Recurrence Risk for Patients With T1,2 Breast Cancer With 1-3 Positive Lymph Nodes Treated With Mastectomy and Systemic Treatment. Int J Radiat Oncol Biol Phys (2014) 89(2):392–8. doi: 10.1016/j.ijrobp.2014.02.013 24721590

[17] Muhsen S Moo TA Patil S Stempel M Powell S Morrow M. Most Breast Cancer Patients With T1-2 Tumors and One to Three Positive Lymph Nodes Do Not Need Postmastectomy Radiotherapy. Ann Surg Oncol (2018) 25(7):1912–20. doi: 10.1245/s10434-018-6422-9 PMC5976529 29564588

[18] Montero A Ciérvide R García-Aranda M Rubio C. Postmastectomy Radiation Therapy in Early Breast Cancer: Utility or Futility? Crit Rev Oncol Hematol (2020) 147:102887. doi: 10.1016/j.critrevonc.2020.102887 32018127

[19] Tang Y Zhang YJ Zhang N Shi M Wen G Cheng J. Nomogram Predicting Survival as a Selection Criterion for Postmastectomy Radiotherapy in Patients With T1 to T2 Breast Cancer With 1 to 3 Positive Lymph Nodes. Cancer (2020) 126 Suppl 16:3857–66. doi: 10.1002/cncr.32963 32710662

[20] Kassak F Rossier C Picardi C Bernier J. Postmastectomy Radiotherapy in T1-2 Patients With One to Three Positive Lymph Nodes - Past, Present and Future. Breast (2019) 48:73–81. doi: 10.1016/j.breast.2019.09.008 31561088

[21] Park HJ Shin KH Kim JH Ahn SD Kim JY Park W. Incorporating Risk Factors to Identify the Indication of Post-Mastectomy Radiotherapy in N1 Breast Cancer Treated With Optimal Systemic Therapy: A Multicenter Analysis in Korea (KROG 14-23). Cancer Res Treat Off J Korean Cancer Assoc (2017) 49(3):739–47. doi: 10.4143/crt.2016.405 PMC5512365 27764904

[22] Wang X Zhang L Zhang X Luo J Wang X Chen X. Impact of Clinical-Pathological Factors on Locoregional Recurrence in Mastectomy Patients With T1-2N1 Breast Cancer: Who Can Omit Adjuvant Radiotherapy? Breast Cancer Res Treat (2021) 190(2):277–86. doi: 10.1007/s10549-021-06378-2 PMC8558198 34490502

[23] Gradishar WJ Moran MS Abraham J Aft R Agnese D Allison KH. NCCN Guidelines® Insights: Breast Cancer, Version 4.2021. J Natl Compr Cancer Netw JNCCN (2021) 19(5):484–93. doi: 10.6004/jnccn.2021.0023 34794122

[24] Velikova G Williams LJ Willis S Dixon JM Loncaster J Hatton M. Quality of Life After Postmastectomy Radiotherapy in Patients With Intermediate-Risk Breast Cancer (SUPREMO): 2-Year Follow-Up Results of a Randomised Controlled Trial. Lancet Oncol (2018) 19(11):1516–29. doi: 10.1016/s1470-2045(18)30515-1 30337220

[25] Kunkler IH Canney P van Tienhoven G Russell NS Group MESTM. Elucidating the Role of Chest Wall Irradiation in 'Intermediate-Risk' Breast Cancer: The MRC/EORTC SUPREMO Trial. Clin Oncol (R Coll Radiol) (2008) 20(1):31–4. doi: 10.1016/j.clon.2007.10.004 18345543

[26] Wei J Jiang Y Shao Z. The Survival Benefit of Postmastectomy Radiotherapy for Breast Cancer Patients With T1-2N1 Disease According to Molecular Subtype. Breast (2020) 51:40–9. doi: 10.1016/j.breast.2020.03.003 PMC7375676 32200207

[27] Goodman CR Seagle BL Kocherginsky M Donnelly ED Shahabi S Strauss JB. 21-Gene Recurrence Score Assay Predicts Benefit of Post-Mastectomy Radiotherapy in T1-2 N1 Breast Cancer. Clin Cancer Res an Off J Am Assoc Cancer Res (2018) 24(16):3878–87. doi: 10.1158/1078-0432.ccr-17-3169 29685878

[28] Zhang WW Tong Q Sun JY Hua X Long ZQ Deng JP. 21-Gene Recurrence Score Assay Could Not Predict Benefit of Post-Mastectomy Radiotherapy in T1-2 N1mic ER-Positive HER2-Negative Breast Cancer. Front Oncol (2019) 9:270. doi: 10.3389/fonc.2019.00270 31041190 PMC6477026

[29] Mamounas EP Liu Q Paik S Baehner FL Tang G Jeong JH. 21-Gene Recurrence Score and Locoregional Recurrence in Node-Positive/ER-Positive Breast Cancer Treated With Chemo-Endocrine Therapy. J Natl Cancer Inst (2017) 109(4):1–8. doi: 10.1093/jnci/djw259 PMC5721938 28122895

[30] Thomssen C Balic M Harbeck N Gnant M. St. Gallen/Vienna 2021: A Brief Summary of the Consensus Discussion on Customizing Therapies for Women With Early Breast Cancer. Breast Care (Basel) (2021) 16(2):135–43. doi: 10.1159/000516114 PMC8089428 34002112

[31] Ohri N Haffty BG. Is There a Role for Postmastectomy Radiation (PMRT) in Patients With T1-2 Tumors and One to Three Positive Lymph Nodes Treated in the Modern Era? Ann Surg Oncol (2018) 25(7):1788–90. doi: 10.1245/s10434-018-6493-7 29700669

