# Supplementary material for: Practical Model to Optimize the Strategy of Adjuvant Postmastectomy Radiotherapy in T1-2N1 Breast Cancer With Modern Systemic Therapy
Source: Front Oncol. 2022 Feb 24;12:789198. doi: 10.3389/fonc.2022.789198 (PMC8908314; doi:10.3389/fonc.2022.789198)
Supplement: Supplementary file 1 [file Table_1.docx]

**Supplemental Table 1:** The univariate and multivariable analyses for Outcomes

| Characteristics |  | | DFS | | | | | | | | | | | | | |
| --- | --- | --- | --- | --- | --- | --- | --- | --- | --- | --- | --- | --- | --- | --- | --- | --- |
|  | High-risk group | | | | | | | |  | Low-risk group | | | | | | |
|  | univariate analyses | | | |  | Multivariable analyses | | |  | univariate analyses | | |  | Multivariable analyses | | |
|  | N of event | 5-y rate | | P value |  | HR | 95% CI | P value |  | N of event | 5-y rate | P value |  | HR | 95% CI | P value |
| **Age (years)** |  |  | | 0.98 |  |  |  |  |  |  |  | 0.69 |  |  |  |  |
| ≤40 | 7 | 88.7 | |  |  |  |  |  |  | 0 | 100.0 |  |  |  |  |  |
| >40 | 32 | 87.3 | |  |  |  |  |  |  | 29 | 92.6 |  |  |  |  |  |
| **Menopausal status** |  |  | | 0.22 |  |  |  |  |  |  |  | 0.27 |  |  |  |  |
| Premenopausal | 13 | 89.9 | |  |  |  |  |  |  | 6 | 96.4 |  |  |  |  |  |
| Postmenopausal | 26 | 85.8 | |  |  |  |  |  |  | 23 | 90.9 |  |  |  |  |  |
| **Tumor size (cm)** |  |  | | 0.52 |  |  |  |  |  |  |  | 0.05 |  |  |  |  |
| ≤2.0 | 8 | 89.0 | |  |  |  |  |  |  | 17 | 94.0 |  |  |  |  |  |
| 2.0-5.0 | 31 | 87.2 | |  |  |  |  |  |  | 12 | 88.9 |  |  |  |  |  |
| **Nuclear grade** |  |  | | 0.12 |  |  |  |  |  |  |  | 0.38 |  |  |  |  |
| Low-Intermediate | 12 | 91.4 | |  |  |  |  |  |  | 21 | 91.9 |  |  |  |  |  |
| High | 23 | 84.0 | |  |  |  |  |  |  | 7 | 91.2 |  |  |  |  |  |
| **Axillary surgery** |  |  | | 0.48 |  |  |  |  |  |  |  | 0.63 |  |  |  |  |
| SLNB alone | 0 | 100.0 | |  |  |  |  |  |  | 1 | 85.7 |  |  |  |  |  |
| ALND | 39 | 87.3 | |  |  |  |  |  |  | 28 | 92.8 |  |  |  |  |  |
| **Number of positive LN** |  |  | | 0.61 |  |  |  |  |  |  |  | 0.53 |  |  |  |  |
| 1-2 | 25 | 87.9 | |  |  |  |  |  |  | 28 | 92.1 |  |  |  |  |  |
| 3 | 14 | 86.6 | |  |  |  |  |  |  | 1 | 100.0 |  |  |  |  |  |
| **ER status** |  |  | | 0.98 |  |  |  |  |  |  |  | 0.60 |  |  |  |  |
| Positive | 27 | 88.5 | |  |  |  |  |  |  | 24 | 93.0 |  |  |  |  |  |
| Negative | 12 | 85.1 | |  |  |  |  |  |  | 5 | 89.8 |  |  |  |  |  |
| **Ki67 index** |  |  | | 0.25 |  |  |  |  |  |  |  | 0.27 |  |  |  |  |
| ≤20% | 2 | 96.0 | |  |  |  |  |  |  | 21 | 91.7 |  |  |  |  |  |
| >20% | 37 | 86.6 | |  |  |  |  |  |  | 8 | 94.1 |  |  |  |  |  |
| **HER2 status** |  |  | | 0.26 |  |  |  |  |  |  |  | 0.43 |  |  |  |  |
| Positive | 16 | 84.1 | |  |  |  |  |  |  | 3 | 97.6 |  |  |  |  |  |
| Negative | 23 | 89.1 | |  |  |  |  |  |  | 26 | 91.7 |  |  |  |  |  |
| **Molecular subtype** |  |  | | 0.51 |  |  |  |  |  |  |  | 0.87 |  |  |  |  |
| Luminal | 27 | 88.5 | |  |  |  |  |  |  | 24 | 93.0 |  |  |  |  |  |
| HER2 positive | 8 | 81.1 | |  |  |  |  |  |  | 2 | 93.8 |  |  |  |  |  |
| Triple negative | 4 | 89.8 | |  |  |  |  |  |  | 3 | 87.2 |  |  |  |  |  |
| **Chemotherapy** |  |  | | <0.01 |  |  |  |  |  |  |  | 0.52 |  |  |  |  |
| Yes | 28 | 89.4 | |  |  | 0.27 | 0.12-0.58 | <0.01 |  | 23 | 92.6 |  |  |  |  |  |
| No | 10 | 66.7 | |  |  | 1 |  |  |  | 6 | 91.5 |  |  |  |  |  |
| **Target therapy** |  |  | | 0.20 |  |  |  |  |  |  |  | 0.82 |  |  |  |  |
| Yes | 11 | 81.3 | |  |  |  |  |  |  | 2 | 100.0 |  |  |  |  |  |
| No | 27 | 89.4 | |  |  |  |  |  |  | 27 | 91.5 |  |  |  |  |  |
| **Endocrine therapy** |  |  | | 0.87 |  |  |  |  |  |  |  | 0.30 |  |  |  |  |
| Yes | 24 | 89.7 | |  |  |  |  |  |  | 21 | 93.3 |  |  |  |  |  |
| No | 14 | 83.6 | |  |  |  |  |  |  | 8 | 88.9 |  |  |  |  |  |
| **PMRT** |  |  | | <0.01 |  |  |  |  |  |  |  | 0.45 |  |  |  |  |
| Yes | 13 | 91.9 | |  |  | 0.50 | 0.24-1.00 | 0.05 |  | 13 | 91.2 |  |  |  |  |  |
| No | 26 | 81.4 | |  |  | 1 |  |  |  | 16 | 93.6 |  |  |  |  |  |

**Supplemental Table 2** Patient and treatment characteristics in the matched cohort after propensity score matching

| Characteristics | Whole matched cohort  （N=392） | No-PMRT  （N=196） | PMRT  （N=196） | P value |
| --- | --- | --- | --- | --- |
| **Age (years)** |  |  |  | 0.71 |
| ≤40 | 31(7.9) | 14(7.1) | 17(8.7) |  |
| >40 | 361(92.1) | 182(92.9) | 179(91.3) |  |
| **Menopausal status** |  |  |  | 0.75 |
| Premenopausal | 144(36.7) | 74(37.8) | 70(35.7) |  |
| Postmenopausal | 248(63.3) | 122(62.2) | 126(63.3) |  |
| **Tumor size (cm)** |  |  |  | 0.13 |
| ≤2.0 | 184(46.9) | 100(51.0) | 84(42.9) |  |
| 2.0-5.0 | 208 (53.1) | 96(49.0) | 112(57.1) |  |
| **Nuclear grade** |  |  |  | 0.34 |
| Low-Intermediate | 200(55.6) | 106(58.2) | 94 (52.8) |  |
| High | 160(44.4) | 76(41.8) | 84(47.2) |  |
| Unknown | 32 | 14 | 18 |  |
| **Number of positive LN** |  |  |  | 0.07 |
| 1-2 | 341(87.0) | 164 (83.7) | 177(90.3) |  |
| 3 | 51(13.0) | 32(16.3) | 19(9.7) |  |
| **ER status** |  |  |  | 0.45 |
| Positive | 311(79.3) | 159(81.1) | 152(77.6) |  |
| Negative | 81(20.7) | 37(18.9) | 44(22.4) |  |
| **Ki67 index** |  |  |  | 0.17 |
| ≤20% | 136(34.7) | 75(38.3) | 61(31.1) |  |
| >20% | 256 (65.3) | 121(61.7) | 135(68.9) |  |
| **HER2 status** |  |  |  | 0.06 |
| Positive | 106(27.0) | 43(21.9) | 63 (32.1) |  |
| Negative | 286(73.0) | 153(78.1) | 133(67.9) |  |
| **Molecular subtype** |  |  |  | 0.06 |
| Luminal | 311(79.3) | 159(81.1) | 152(77.6) |  |
| HER2 positive | 46(11.7) | 16(8.2) | 30(15.3) |  |
| Triple negative | 35(8.9) | 21(10.7) | 14(7.1) |  |
| **Chemotherapy** |  |  |  | 0.85 |
| Yes | 362(92.3) | 182(92.9) | 180(91.8) |  |
| No | 30(7.7) | 14(7.1) | 16(8.2) |  |
